# Wound healing activity and flavonoid contents of purslane (*Portulaca grandiflora*) of various varieties

**DOI:** 10.1039/d3ra00868a

**Published:** 2023-03-28

**Authors:** Antonius Budiawan, Agus Purwanto, Levi Puradewa, Erlien Dwi Cahyani, Christiana Endang Purwaningsih

**Affiliations:** a Pharmacy Diploma III Department, Widya Mandala Surabaya Catholic University Manggis 15-17 Madiun City 63131 East Java Indonesia antonius.budiawan@ukwms.ac.id; b Biology Department, Widya Mandala Surabaya Catholic University Manggis 15-17 Madiun City 63131 East Java Indonesia; c Pharmacy Diploma III Department, Widya Mandala Surabaya Catholic University Manggis 15-17 Madiun City 63131 East Java Indonesia; d Pharmacy Diploma III Department, Widya Mandala Surabaya Catholic University Manggis 15-17 Madiun City 63131 East Java Indonesia; e Biology Department, Widya Mandala Surabaya Catholic University Manggis 15-17 Madiun City 63131 East Java Indonesia

## Abstract

Purslane has various varieties with different active metabolite contents that need to be explored further to find each variety's activity in wound healing. Different purslane herbs showed different antioxidant activities, suggesting they will have different flavonoid content and wound healing activity. This research aimed to evaluate purslane's total flavonoid content and wound-healing activity. The wounds induced on the rabbit back skin were divided into 6 treatment groups such as negative control, positive control, 10 and 20% purslane herbs extract varieties A, and 10 and 20% purslane herbs extract varieties C. Wounds were treated twice daily for 2 weeks, and measured on day 0, 7, 11, and 14. Total flavonoid content was measured with the AlCl_3_ colorimetric method. The wounds treated with 10 and 20% purslane herbs extract varieties A (*Portulaca grandiflora* magenta flower) have 0.32 ± 0.55 and 1.63 ± 1.96 mm wound diameters on day 7 and healed on day 11. The wounds treated with 10 and 20% purslane herbs extract varieties C (*Portulaca grandiflora* pink flower) showed 2.88 ± 0.51 and 0.84 ± 1.45 mm diameter and healed on day 11. The purslane herb A showed the highest wound healing activity, and purslane varieties A and C total flavonoid contents were 0.55 ± 0.02 and 1.58 ± 0.02% w/w, respectively.

## Abstract 2

Different flower colour varieties of purslane (*Portulaca grandiflora*) showed different total flavonoid content. Flavonoids have a main role in wound healing activity in rabbit skin as antibacterial, antiinflammation, and antioxidant agents.

## Graphical abstract

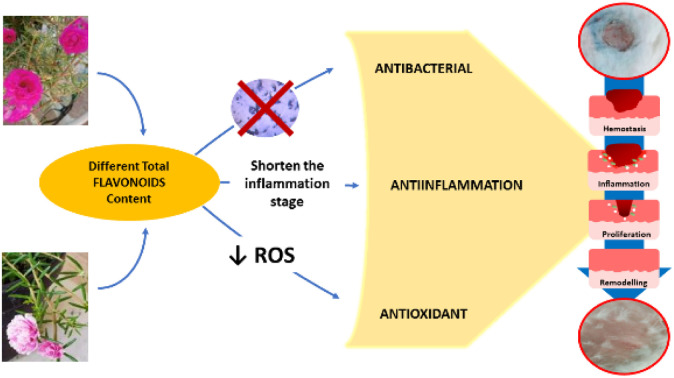

## Introduction

A wound is an injury on the outside body part tissue that disturbs physical function.^1,2^ Approximately 1% of the world's population is believed to be affected by wounds.^3^ Meanwhile, the prevalence of wounds in Indonesia based on the proportion of injuries is 20%.^4^ Indonesia is an archipelagic country prone to natural disasters and has many remote areas. Wound care will be a big problem for people living in remote areas with limited wound treatment access. Wounds not appropriately treated will cause disability or at least scars that cause discomfort.^5^

Wound healing at the molecular level is a complex process involving various components.^6^ These components include mediator cells and extracellular matrix. The stages of wound healing itself are divided into four stages: hemostasis, inflammation, proliferation, and remodeling.^7^ When skin gets injured, the hemostasis stage starts and follows by an increase in platelet aggregation and the production of various chemotactic factors such as TGF-β1, TGF-β2, and PDGF.^8^ Inflammatory cells such as neutrophils, lymphocytes, and macrophages at the inflammatory stage will go to the wound's center to prevent infection. In the next stage (proliferation and remodeling), keratinocytes, macrophages, platelets, and endothelial cells in the wound area release various growth factors so that new cells are formed in the wound area.^9^

Expensive and unaffordable treatment costs often hamper wound healing. So wound healing therapy at a low cost is needed at this time. Medicinal plants are sources that come from nature, so they have minimal side effects and are cheaper, so they can be a safe alternative to synthetic drugs for wound treatment.^10^ Several medicinal plants have been investigated for their healing properties; one is purslane herbs.

Purslane herbs (*Portulaca grandiflora*) is a plant that grows well in a tropical climate. Society mainly considers it a problematic plant. In addition, purslane herbs are only used as decorative plants in the front yard. This fact encourages the researchers to find other potencies that could be utilized from purslane herbs. Several studies have been conducted to find purslane efficacies on diseases, and one of these efficacies is antibacterial activity.^11^ This antibacterial activity of purslane herbs can accelerate wound healing and have the potency to be developed as wound treatment topical products.

Purslane has various varieties with different active metabolite contents that need further exploration to find each variety's activity in wound healing.^12^ Based on several studies, total phenol and antioxidant activity from different flower colour varieties showed different results.^13^ Flavonoid is a secondary metabolite widely known for its antioxidant properties.^14^ Different flower colour varieties of purslane herbs showed different antioxidant activities, suggesting they will have different flavonoid content and wound healing activity. Based on this background, research will be needed to determine purslane's various total flavonoid content and wound healing activity.

## Result and discussion

### Purslane herbs (*Portulaca grandiflora*) determination result

The first step of the research was the purslane herbs determination process. This step was performed to ensure the sample was the correct purslane (*Portulaca grandiflora*). The determination process was performed on purslane herbs A and C. The determination process was performed by direct morphology observation based on determination keys and observation reports.^18–23^

The observation result showed that two varieties of purslane (*Portulaca grandiflora*) have the characteristics as follows: magenta and pink colored rose-like flowers, succulent plant, 15–30 cm high stem, round and solid stem, redness brown colored stem skin with slight hair on the surface, slippery surface, upright or fall growing direction, and branching is forming from the base point characteristic.

**Fig. 1:**
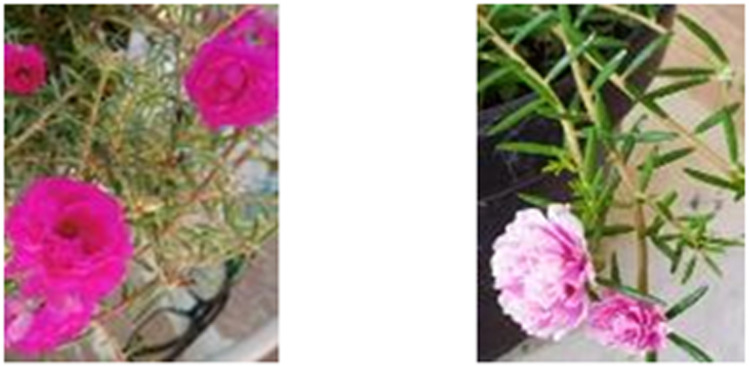
The branch, leaves, and flower morphology of purslane herbs (*Portulaca grandiflora*).

The leaf is single thick, and meaty, 2–5 cm long, shiny green colored, shipshape spread and spiral structure, linier-subulatus or cylindrical shape, with sharp (*acutus*) tip and blunt (*obtusus*) pole, flat (*integer*) side with 1–3 cm long. The flower is bisexual, single complex, and blooms on the end of the branch with a round (*obovatus*) rose-like shape, the diameter is 25–35 mm, and the stamens are orange-colored with more than 20–50 stamen count. The flower pole is surrounded by 8–10 leaves, 2 petals, and the largest flower could reach 4 cm in diameter. The morphological observation is similar to the previous researcher's report.^22^ The different *Portulaca grandiflora* variety morphology that has been used in this research was on the flower color, which was magenta (variety A) and pink with white fade (variety C) color (Fig. 1).

### Extraction result

The purslane herbs extraction used a simple maceration method and fit with a thermolabile active compound. Ethanol was used as an extraction solvent because it could give a higher extract yield and total phenol.^13^ Purslane herbs C showed a higher extract yield of 10.99% compared to purslane herbs A with 9.49%.

### Wound healing activity test result

The rabbit skin has a similar aging process, wound healing time, and response to drugs to humans so it can be used as a wound-healing activity animal test.^16^ In addition, rabbits have broad back skin, making it easier to observe wounds visually. The wound healing test was started with wound induction. The hair on the back part of the rabbit was shaved with shaving tools to simplify wound induction and wounds diameter observation. Lidocaine injection was used as anesthesia because of its good local activity during the wound induction process.

The wound-healing activity test result (Table 1) based on wound diameter size on day 7 showed that purslane herbs A extract 10% concentration and purslane herbs C extract 20% concentration groups have significantly different (*p* < 0.05) from the negative control group. A10 also showed significant differences (*p* < 0.05) with other groups except for A20 and C20. The A10 group showed the smallest wound diameter average with 0.32 ± 0.55 mm size. The negative control group showed the largest wound diameter average with 6.90 ± 1.03 mm size.

**Table 1:** Wounds diameter on days 0, 7, 11, and 14

Groups	Wound diameter size on the day – (mm)			
	0	7	11	14
K(−)	8.00 ± 0.00	6.90 ± 1.03^b^^,^^c^^,^^d^^,^^e^^,^^f^	4.75 ± 0.24^b^^,^^c^^,^^d^^,^^e^^,^^f^	0.94 ± 0.01
K(+)	8.00 ± 0.00	3.03 ± 0.63^a^	0.00 ± 0.00^a^	—
A10	8.00 ± 0.00	0.32 ± 0.55^a^^,^^b^^,^^e^	0.00 ± 0.00^a^	—
A20	8.00 ± 0.00	1.63 ± 1.96^a^	0.00 ± 0.00^a^	—
C10	8.00 ± 0.00	2.88 ± 0.51^a^^,^^c^	0.00 ± 0.00^a^	—
C20	8.00 ± 0.00	0.84 ± 1.45^a^^,^^b^^,^^d^	0.00 ± 0.00^a^	—

a Significantly different from K(−) (negative control).

b Significantly different from K(+) (positive control).

c Significantly different from A10 (purslane herbs A extract 10% concentration).

d Significantly different from A20 (purslane herbs A extract 20% concentration).

e Significantly different from C10 (purslane herbs C extract 10% concentration).

f Significantly different from C20 (purslane herbs C extract 20% concentration).

The wound diameter observation on day 11 significantly differed between the negative control group and other groups. The negative control group showed the largest size wound diameter average with 4.75 ± 0.24 mm size. On this day, all the groups besides the negative control group showed complete wound recovery.

The wounds treated with distilled water were still not fully recovered on day 14. The wound diameter average was measured at 0.94 ± 0.01 mm. The different results showed by betadine solution and purslane herbs extract-treated groups that were fully healed since day 11.

**Fig. 2:**
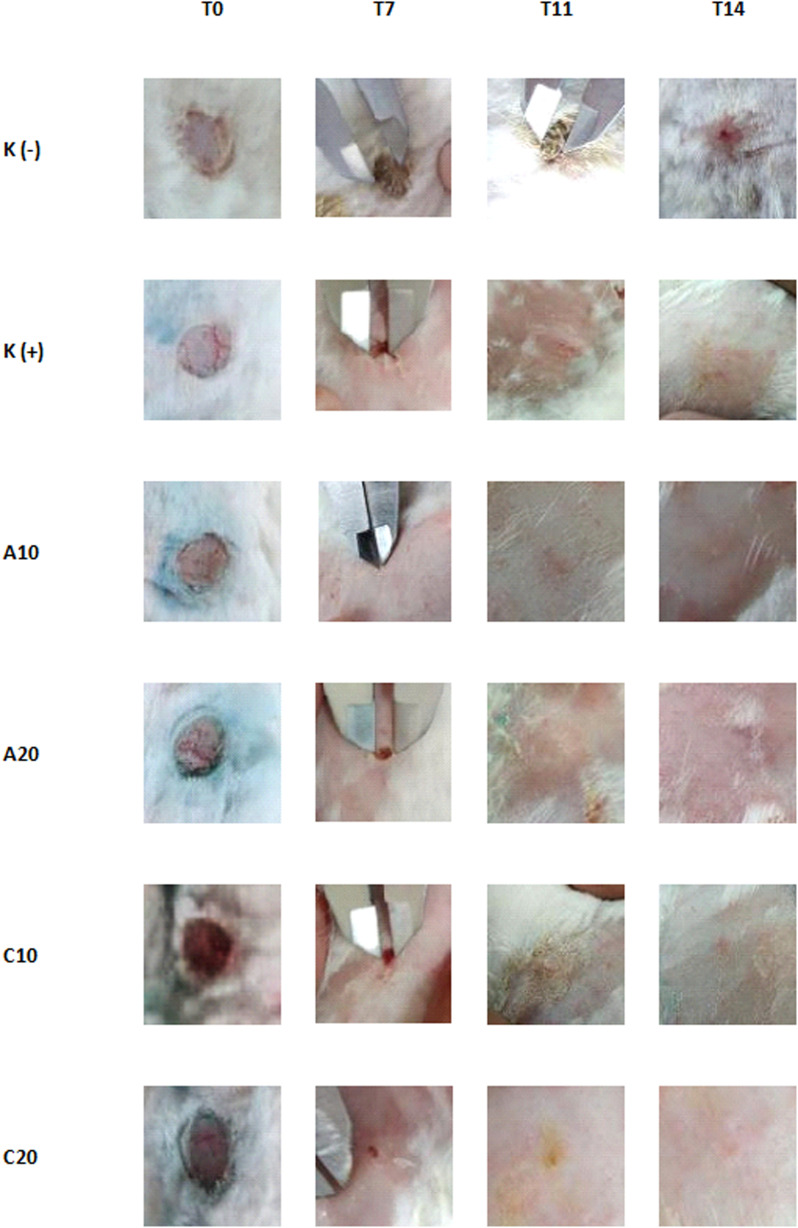
Wounds visualization on days 0, 7, 11, and 14.

The wound healing activity was shown by betadine solution and extract-treated groups. The activity was shown by wound diameter changes that were smaller daily and healed starting from day 11 to fully recover on day 14 (Fig. 2).

The different healing process was shown by the negative control group that was treated with distilled water. The wounds showed slow healing progress. The healing progressed until the end of observation, and the wounds were still not fully closed or recovered on day 14. Unfortunately, the observation was not prolonged after day 14, so the real healing timing from the negative control group was never established. However, the wounds would be healed naturally on days 13–16, depending on the size.^16^

The betadine solution-treated wounds showed better healing results compared to the untreated wounds. Betadine solution contains povidone-iodine 10%, an antibacterial compound widely used as first aid to treat wounds. Povidone-iodine is an iodoform compound that penetrates inside bacteria cells and oxidizes key protein, nucleotide, and fat acid bacteria leading to bacteria death.^24^ Without bacteria around, the wound will be healed faster because the inflammation stage of the healing process will end faster. The proliferation and remodeling will be achieved even faster rather than untreated wounds. The inflammation process is needed to transfer neutrophils, monocytes, and lymphocytes to the wound site to kill invading bacteria. This process will be prolonged if bacteria are too many to handle, so antibacterial compounds such as povidone-iodine are needed to accelerate the healing process.^25^

The wound diameter on all purslane herbs extracts groups showed a smaller size daily and fully recovered on day 14, similar to the betadine solution-treated group. The different concentration dosages on purslane herbs A and C extract groups showed different diameter sizes even though the differences were insignificant (*p* > 0.05). It may be concluded that different concentration dosages did not give different wound healing activities. However, the dosage variation in this research is only 10% and 20%, and the effect of broader and higher dosage concentrations on wound healing activity remains unknown.

### Qualitative phytochemical screening result

Phytochemical screening analysis showed that purslane herbs variety A and C extract showed similar compounds: alkaloids, flavonoids, saponins, and tannins. Alkaloid reaction test results showed brown sediment. Furthermore, on saponin reaction test showed persistent foam on HCl addition. The color-changing to dark green showed all extracts after FeCl_3_ addition showed tannins content. Brown sediment was shown after chloroform, and H_2_SO_4_ concentrated addition showed terpenoid contains. The result was comparable with other studies, which showed that ethanolic purslane herbs extract contains flavonoids, alkaloids, terpenoids, saponins, and tannins^26^ (Table 2).

**Table 2:** Purslane herbs of various varieties extract phytochemical screening result

Extract	Flavonoid	Alkaloid	Terpenoid	Saponin	Tannin
Purslane herbs A	+	+	+	+	+
Purslane herbs C	+	+	+	+	+

### Total flavonoid content analysis result

The calibration curve determination result gives the formula *y* = 00 233 + 002 77*x* with a 0.9971*r* value. The higher flavonoid content was shown by purslane herbs C extract with 1.58 ± 0.02%. The purslane herbs A extract showed smaller flavonoid content with 0.55 ± 0.02%. Those flavonoid contents were significantly different, with a *p*-value < 0.05. This result showed that different varieties contain different metabolite content. This is comparable with other research that showed different flavonoid content in the various purslane varieties.^12^

### Discussion

The determination result showed that purslane herb A is *Portulaca grandiflora* with a magenta-colored flower variety. Moreover, purslane herb C is *Portulaca grandiflora* with a pink-colored flower variety. These differences are related to secondary metabolites compound content in the purslane herbs.^12^ Those purslane varieties have shown antibacterial activity on *Staphylococcus aureus*, *Escherichia coli*, and *Pseudomonas aeruginosa*.^15^ Those bacteria are commonly found in wound infections, so this extract has the potency to be developed as a wound-healing compound.

A wound is an injury that could be acquired on every body part. The open wound on the skin will increase bacteria infection risk because the skin contains many bacteria. This open wound condition will increase bacteria's chance to enter and invade the deeper tissue.^1^ The body's inflammation mechanism response will follow this bacteria invasion. The pro-inflammation agents such as neutrophils, macrophages, and lymphocytes will migrate into wound site and kill the bacteria that is invading with various mechanisms such as phagocytes and apoptosis.^25^ This process will be prolonged if bacteria are too many to handle, so an active compound with antibacterial activity is necessary to shorten this stage. The inflammation stage will also be prolonged by oxidative stress caused by free radical excessive accumulation.^27^ This condition will cause further cell obstruction, prolonging the wound-healing process. That is why the active compound with antioxidant activity also accelerates the wound healing process.

Based on phytochemical screening results, ethanolic purslane herbs extract contains various secondary metabolites such as flavonoids, alkaloids, saponin, tannins, and terpenoids that have taken part in wound healing activity with various mechanisms^28^ that were shown by wound healing activity results. The wounds treated with purslane herbs extract A and C healed with no significantly different from betadine solution-treated wounds, especially on days 11 and 14. At the end of observation, all purslane extract-treated wounds showed a similar healed stage to betadine solution-treated wounds. Different purslane species (*Portulaca oleracea*) grown in Jordania showed similar results with different animal tests.^29^

Flavonoid content in purslane herb extracts is known for its antiinflammation, antibacterial, and antioxidant activity.^30,31^ As an antiinflammation, flavonoids shorten the inflammation stage, leading to the proliferation and remodeling stage, shortening the healing process. The antibacterial activity of flavonoids supports this activity. The inflammation process ends faster in line with the bacteria number decline. Flavonoid antibacterial activity refers to its ability to complex forming with bacteria cell membrane protein.^32^ Flavonoids work as an antioxidant by scavenging free radicals that lead to oxidative stress prevention.^27^ Free radical is a reactive molecule with antibacterial properties in a reactive oxygen species (ROS) form.

Nevertheless, in a saturated condition, ROS could cause further tissue damage at the wound site.^33,34^ Thus condition prolonged the healing process, so the antioxidant activity of flavonoids in purslane herb extracts has the benefit that could prevent oxidative stress from happening.^14,34,35^ Flavonoids could donate their electrons to ROS, preventing ROS from taking electrons from molecules to tissue formation, such as DNA, protein, and lipids.^33^

Total flavonoid content analysis showed that purslane herb extracts of both varieties showed flavonoid content even though this result did not align with the wound healing activity test. Purslane herb variety A extract showed higher wound healing activity than variety C extract, even though its flavonoid content was lower than purslane herb variety C extract. This may be caused by differences in other metabolite constituents in both purslane herb varieties.

Another constituent in the purslane herb suspected of its role in wound healing is an alkaloid. Alkaloids may accelerate wound healing and tissue granulation by increasing collagen and fibroblast and lowering inflammation cell numbers.^36^ Fibroblast is a connective tissue that plays in collagen production.^37^ Collagen is a connective protein responsible for elasticity and skin structure strength.^38,39^ Alkaloids also play a role in SRC/MEK/ERK signaling pathway regulation that promotes the wound healing process.^40^

Saponin detected in purslane herb A, and C varieties is a secondary metabolite with antibacterial activity. This activity is related to its ability to increase bacteria cell membrane permeability, leading to hemolysis.^41^ Other than that, saponin may also increase collagen production and accelerate cell epithelization in the wound.^42^ Saponin also had an essential role in high glucose-induced wound healing by increasing endothelial cell proliferation.^43^

Tannins may also play an essential role in the wound-healing process. Tannins are known for their antibacterial activity in rebuilding damaged tissue and accelerating fibrous tissue contraction in wound healing.^44^ Fibrous contraction by fibroblast will trigger scar formation that protects cell formation in the wound site. Tannin's antibacterial activity may be caused by its toxicity to the microorganism related to bacteria enzyme and substrate complex formation.^45^ Other than this mechanism, tannins are also known for their antioxidant with similar free radical scavenging mechanism activity to flavonoids.^46,47^

## Experimental section

### Materials

Tools to conduct *in vivo* wound healing activity tests were scissors and razors to cut the rabbit hair, 1 ml disposable syringe, biopsy punch 8 mm diameter, anatomical forceps, scalpel blade, vernier calipers to measure wound diameter, and vacuum rotary evaporator. Analytical balance, spectrophotometer UV-vis, and glassware were used to perform phytochemical and total flavonoid content analysis.

The sample of two varieties of purslane was obtained from Madiun City of Java Island in Indonesia. The solvent that was used in the extraction process was ethanol 70%. The New Zealand rabbit was used as an animal test to perform wound healing activity. Lidocaine injection was used as a local anesthetic on rabbit skin when wound excision was conducted. Betadine solution and distilled water were used as the positive and negative control, respectively. The materials used for phytochemical screening and total flavonoid content analysis were ethanol 96% pro analysis, AlCl_3_, potassium acetate, and distilled water.

### Methods

#### Purslane herbs characterization (*Portulaca grandiflora*)

Two kinds of purslane herbs obtained from Madiun City were characterized to know their variety. Determination was conducted in Biology Laboratory, Biology Department, Widya Mandala Surabaya Catholic University.

#### Extraction process

Two hundred grams of each dried purslane herbs variety were macerated with 500 ml ethanol 70% for 5 days. The solid residue from the maceration process was macerated once more with the same amount of solvent. The first and second macerates were mixed and evaporated with a vacuum rotary evaporator at 50 °C until a thick extract was obtained. The thick extract was used for phytochemical screening, total flavonoid content analysis, and *in vivo* wound healing activity tests.

#### Wound healing activity test

The rabbit was used as an animal test to conduct *in vivo* wound healing activity. The New Zealand rabbit used in this study was 3–5 months old and had a 1.5–2.5 kg weight. Ethical clearance was obtained from Health Research Ethics Committee Faculty of Medicine, Muhammadiyah Surakarta University, Indonesia.

Rabbit's hair on the back part was shaved with shaving tools. The rabbit skin was anesthetized with lidocaine with 0.0125–0.7600 ml per kg BW dose until showing unresponsive when stabbed with a needle. A biopsy punch of 8 mm diameter was used to make wound excision on rabbit skin. Three rabbits were induced with six wounds for each rabbit.

The wounds on the rabbit's back skin were grouped into 6 treatment groups such as negative control (distilled water), positive control (betadine solution), 10 and 20% purslane herbs extract varieties A, 10 and 20% purslane herbs extract varieties C.

The wounds were cleaned and disinfected with an antiseptic solution and waited until the blood stopped before the treatment was applied twice daily for two weeks. The macroscopic condition observation and wound diameter measurement was conducted on days 0, 7, 11, and 14.

#### Qualitative phytochemical screening

The flavonoid identification was conducted by dissolving 0.5 grams of each various variety of purslane ethanolic extract in the distilled water and added with magnesium and 5 drops of HCl 2 N. The mixture was heated for 5–10 minutes. After filtration and cooling, the filtrate was added with amyl alcohol and shaken vigorously. The reaction result is positive if red color is formed in the amyl alcohol layer.^17^

The alkaloid identification was conducted by 0.5 grams of each various variety of purslane ethanolic extract was added with 1 ml ammonia and then added with chloroform. The mixture was filtrated, and the filtrate was placed in the test tube and added with 2 N HCl. The mixture was shaken and waited until separation occurred. The filtrate was divided into three parts in the different test tubes. One drop of Dragendorff reagent was added to the first filtrate. The reaction result is positive if brown sediment is formed. One drop of Mayer reagent was added to the second filtrate, and the reaction was positive if white sediment formed. The third filtrate is blank or negative control.^17^

The saponin identification was conducted by placing 0.5 grams of each various variety of purslane ethanolic extract into a test tube and then dissolved with hot distilled water. After the mixture was cold, it was shaken for 10 seconds. The reaction is positive if 1–10 cm persistent foam is formed for 10 minutes, and stays still after adding 1–2 drops of 2 N HCl.^17^

The terpenoid identification was conducted by placing 0.5 grams of each various variety of purslane ethanolic extract into a test tube and then added with chloroform and H_2_SO_4_ concentrate. The reaction is positive if brown sediment is formed.

The tannin identification was conducted by placing 0.5 grams of each various variety of purslane ethanolic extract into a test tube and then added with 1% FeCl_3_ solvent. The reaction is positive if green or dark blue color is formed.

The total flavonoid content of various varieties of purslane was measured with AlCl_3_ colorimetric methods. Twenty-five mg of quercetin was dissolved with 25 ml ethanol in a 25 ml volumetric flask to obtain a 1000 μg ml^−1^ initial solution. Standard solutions of 10, 20, 40, 60, 80, and 100 ppm were made from the initial solution. Each standard solution was pipetted 0.5 ml and then was added with 1.5 ml ethanol 96%, 1 ml 10% AlCl_3_, 1 ml potassium acetate 1 M, and 2.8 ml distilled water. The solution was incubated at 25 °C for 30 minutes. The absorbance was measured with a spectrophotometer UV-vis on a wavelength (*λ*) of 434.2 nm. The absorbance reading result was used to make a calibration curve of absorbance *vs.* quercetin concentration.

Five grams of each various variety of purslane ethanolic extract was dissolved with 25 ml ethanol and stirred at 200 rpm, and then filtrated. The filtrate was added to 25 ml ethanol. The blank solution for the flavonoid content assay was made from 0.5 ml ethanol that was added with 1.5 ml ethanol 96%, 1 ml 10% AlCl_3_, 1 ml potassium acetate 1 M, and 2.8 ml distilled water. One ml of various purslane ethanolic extract was added with ethanol until the marking sign of the 10 ml volumetric flask. Half ml of the solution was pipetted and added with 1.5 ml ethanol 96%, 1 ml 10% AlCl_3_, 1 ml potassium acetate 1 M, and 2.8 ml distilled water. The mixture was incubated at 25 °C for 30 minutes, and the absorbance was read with a spectrophotometer UV-vis on *λ* 434.2 nm. The total flavonoid content was counted with the following equation:*F* = (*c* × *V* × *f* × 10 − 6)/*m* × 100%Note: *F* = flavonoid content, *c* = quercetin equality (g ml^−1^), *V* = total extract volume, *f* = dilution factor, *m* = sample weight (g).

#### Data analysis

The observed data were statistically analyzed with a 95% (*p* < 0.05) confidence limit using the Kruskal–Wallis analysis test.

### Live subject statement

All animal procedures were performed under the guidelines for care and use of laboratory animals of Widya Mandala Surabaya Catholic University, Indonesia, and approved by the Health Research Ethics Committee Faculty of Medicine, Muhammadiyah Surakarta University, Indonesia.

## Conclusions

Two varieties of purslane herbs (magenta and pink flower) showed different wound healing activities, especially on day 7. The wound healing activity test of purslane herbs A (*Portulaca grandiflora* magenta flower variety) 10 and 20% concentration showed 0.32 ± 0.55 and 1.63 ± 1.96 mm, respectively, wound diameter size. The purslane herbs C (*Portulaca grandiflora* pink flower variety) 10 and 20% concentration showed 2.88 ± 0.51 and 0.84 ± 1.45 mm, respectively, wound diameter size. The purslane herbs A (*Portulaca grandiflora* magenta flower variety) 10 and 20% concentrations showed higher activity in the wound healing process on day 7 with 0.32 ± 0.55 and 1.63 ± 1.96 mm, respectively, which is the smallest wound diameter. Purslane herb varieties A and C total flavonoid contents were 0.55 ± 0.02 and 1.58 ± 0.02% w/w, respectively.

## Author contributions

Experimental work (AB, LP, AP, EDC), data analysis (AB and CEP), and writing the manuscript (AB and AP).

## Conflicts of interest

There are no conflicts to declare.

## Supplementary Material

## References

[cit1] Price S. A. and Wilson L. M., Patofisiologi: Konsep Klinis Proses-proses Penyakit, 6th edn, 2006

[cit2] Naseri E., Ahmadi A. (2022). Eur. Polym. J..

[cit3] Grover P., Khanna K., Bhatnagar A., Purkayastha J. (2021). Biomed. Pharmacother..

[cit4] The Ministry of Health Republic of Indonesia , Laporan Nasional Riskesdas, 2018

[cit5] Zhang L., Tai Y., Liu X., Liu Y., Yang C., Kong D., Qi C., Wang S., Midgley A. C. (2021). Appl. Mater. Today.

[cit6] Patel S., Srivastava S., Singh M. R., Singh D. (2019). Biomed. Pharmacother..

[cit7] Low J. S., Mak K. K., Zhang S., Pichika M. R., Marappan P., Mohandas K., Balijepali M. K. (2021). Fitoterapia.

[cit8] Assar D. H., Elhabashi N., Mokhbatly A. A. A., Ragab A. E., Elbialy Z. I., Rizk S. A., Albalawi A. E., Althobaiti N. A., Jaouni S. A., Atiba A. (2021). Biomed. Pharmacother..

[cit9] Okur M. E., Karantas I. D., Şenyiğit Z., Okur Ü. N., Siafaka P. I. (2020). Asian J. Pharm. Sci..

[cit10] Mssillou I., Bakour M., Slighoua M., Laaroussi H., Saghrouchni H., Amrati F. E., Lyoussi B., Derwich E. (2022). J. Ethnopharmacol..

[cit11] Fung K. P., Han Q. B., Ip M., Yang X. S., Lau C. Bs., Chan B. Cl. (2017). Hong Kong Med. J..

[cit12] Alam Md A., Juraimi A. S., Rafii M. Y., Abdul Hamid A., Aslani F., Hasan M. M., Zainudin M. A. M., Uddin Md. K. (2014). BioMed Res. Int..

[cit13] Lim C. K., Tiong W. N., Loo J. L. (2014). Int. J. Phytopharm..

[cit14] Carvalho M. T. B., Araújo-Filho H. G., Barreto A. S., Quintans-Júnior L. J., Quintans J. S. S., Barreto R. S. S. (2021). Phytomedicine.

[cit15] Purwanto A. (2021). Agri-Tek: Jurnal Ilmu Pertanian, Kehutanan dan Agroteknologi.

[cit16] Grada A., Mervis J., Falanga V. (2018). J. Invest. Dermatol..

[cit17] The Ministry of Health Republic of Indonesia , Materia Medika Indonesia V, 1989

[cit18] Van Steenis C. G. G. J. , Flora, 2008

[cit19] Uddin M. K., Juraimi A. S., Hossain Md. S., Nahar M. A. U., Ali Md. E., Rahman M. M. (2014). Sci. World J..

[cit20] Setyowati H. (2017). Cont. Prof. Dev..

[cit21] Djauhariya E. and Hernani H., Gulma Berkhasiat Obat, 2004

[cit22] Setiawan F. I. D., Aisyah S. I., Krisantini K. (2016). Journal of Tropical Crop Science.

[cit23] Setyawati T. , Narulita S., Bahri I. P. and Raharjo G. T., A Guide Book to Invasive Plant Species in Indonesia, 2015

[cit24] Lepelletier D., Maillard J. Y., Pozzetto B., Simon A. (2020). Antimicrob. Agents Chemother..

[cit25] Guo S., Di Pietro L. A. (2010). J. Dent. Res..

[cit26] Londonkar R., Nayaka H. (2011). J. Pharma Res..

[cit27] Fitzmaurice S. D., Sivamani R. K., Isseroff R. R. (2011). Skin Pharmacol. Physiol..

[cit28] Adiele L. C., Adiele R. C., Enye J. C. (2014). Asian Pac. J. Trop. Med..

[cit29] Rashed A. N., Afifi F. U., Disi A. M. (2003). J. Ethnopharmacol..

[cit30] Zhou Y. X., Xin H. L., Rahman K., Wang S. J., Peng C., Zhang H. (2015). BioMed Res. Int..

[cit31] Masoodi M. H., Ahmad B., Mir S. R., Zargar B. A., Tabasum N. (2011). J. Pharm. Res..

[cit32] Royani A., Hanafi M., Julistiono H., Manaf A. (2023). Mater. Today: Proc..

[cit33] Dunnill C., Patton T., Brennan J., Barrett J., Dryden M., Cooke J., Leaper D., Georgopoulos N. T. (2017). Int. Wound J..

[cit34] Priyadarshi A., Keshri G. K., Gupta A. (2022). Phytomedicine Plus.

[cit35] Asante-Kwatia E., Evelyn A., Silas J., Yakubu G., Lord G. A. H., Adjei-Hinneh G., Amponsah I. K., Mensah A. Y. (2021). Heliyon.

[cit36] Nagappan T., Segaran T. C., Wahid M. E. A., Ramasamy P., Vairappan C. S. (2012). Molecules.

[cit37] Bao L., Cai X., Zhang M., Xiao Y., Jin J., Qin T., Li Y. (2022). J. Funct. Foods.

[cit38] Bainbridge P. (2013). J. Wound Care.

[cit39] Sharma S., Rai V. K., Narang R. K., Markandeywar T. S. (2022). Life Sci..

[cit40] Shi X. Q., Chen G., Tan J. Q., Li Z., Chen S. M., He J. H., Zhang L., Xu H. X. (2022). J. Ethnopharmacol..

[cit41] Wei M. P., Yu H., Guo Y. H., Cheng Y. L., Xie Y. F., Yao W. R. (2021). Food Control.

[cit42] MacKay D., Miller A. L. (2003). Alternative Med. Rev..

[cit43] Lei T., Gao Y., Duan Y., Cui C., Zhang L., Si M. (2022). Environ. Toxicol..

[cit44] Santana A., Barros A., Oliveira H., Victor I. (2017). Biomed. Pharmacother..

[cit45] Scalbert A. (1991). Phytochemistry.

[cit46] Heinonen M. (2007). Mol. Nutr. Food Res..

[cit47] González C. M., Llorca E., Quiles A., Hernando I., Moraga G. (2022). LWT–Food Sci. Technol..

